# Dehydrogenation of Ethylene on Supported Palladium Nanoparticles: A Double View from Metal and Hydrocarbon Sides

**DOI:** 10.3390/nano10091643

**Published:** 2020-08-21

**Authors:** Oleg A. Usoltsev, Anna Yu. Pnevskaya, Elizaveta G. Kamyshova, Andrei A. Tereshchenko, Alina A. Skorynina, Wei Zhang, Tao Yao, Aram L. Bugaev, Alexander V. Soldatov

**Affiliations:** 1 The Smart Materials Research Institute, Southern Federal University, 178/24 Sladkova, 344090 Rostov-on-Don, Russia; oleg-usol@yandex.ru (O.A.U.); annpnevskaya@yandex.ru (A.Y.P.); kamyshova.liza@gmail.com (E.G.K.); tereshch1@gmail.com (A.A.T.); alinaskorynina@gmail.com (A.A.S.); soldatov@sfedu.ru (A.V.S.); 2 National Synchrotron Radiation Laboratory, University of Science and Technology of China, Hefei 230029, China; zwei2319@mail.ustc.edu.cn (W.Z.); yaot@ustc.edu.cn (T.Y.)

**Keywords:** palladium nanoparticles, ethylene dehydrogenation, XANES, EXAFS, DRIFTS, DFT

## Abstract

Adsorption of ethylene on palladium, a key step in various catalytic reactions, may result in a variety of surface-adsorbed species and formation of palladium carbides, especially under industrially relevant pressures and temperatures. Therefore, the application of both surface and bulk sensitive techniques under reaction conditions is important for a comprehensive understanding of ethylene interaction with Pd-catalyst. In this work, we apply in situ X-ray absorption spectroscopy, X-ray diffraction and infrared spectroscopy to follow the evolution of the bulk and surface structure of an industrial catalysts consisting of 2.6 nm supported palladium nanoparticles upon exposure to ethylene under atmospheric pressure at 50 °C. Experimental results were complemented by ab initio simulations of atomic structure, X-ray absorption spectra and vibrational spectra. The adsorbed ethylene was shown to dehydrogenate to C_2_H_3_, C_2_H_2_ and C_2_H species, and to finally decompose to palladium carbide. Thus, this study reveals the evolution pathway of ethylene on industrial Pd-catalyst under atmospheric pressure at moderate temperatures, and provides a conceptual framework for the experimental and theoretical investigation of palladium-based systems, in which both surface and bulk structures exhibit a dynamic nature under reaction conditions.

## 1. Introduction

Palladium catalysts are being extensively used for hydrogenation of unsaturated hydrocarbons (e.g., selective hydrogenation of acetylene traces in ethylene-rich mixtures) [1,2]; a large number of studies was aimed to get structural insights into the catalytic processes [3,4,5,6,7,8,9,10]. An important step in such reactions is the adsorption of hydrocarbon molecules on the palladium surface, since different adsorption geometries can lead to different reaction products [1].

In contrast to the adsorption of ethylene and other hydrocarbons over platinum extensively examined by the group of Zaera [11,12,13] and numerous other groups [8,9,12,14,15,16,17,18,19,20,21], less attention has been paid to ethylene on palladium [8,9,15,17,20,21,22,23,24]. In most of these studies, infrared spectroscopy is considered as a main source of information due to sensitivity to surface adsorbed species. Density functional theory (DFT) was also widely applied to obtain the structure of intermediate species; however, only a few studies exploited DFT to go beyond the qualitative analysis of infrared spectra towards their theoretical modeling [18,25,26,27,28]. For both Pd and Pt surfaces, π- and di-*σ*-bonded ethylene was reported, with further transformation to ethylidyne which was considered as an important intermediate in ethylene hydrogenation reaction [22]. The possibility of ethylidyne layers formation over the Pd (111) surface at high pressure was also shown [24]. In the recent works of Bowker [29,30,31], it was highlighted that under industrially relevant pressures on real catalysts, the processes are different from that on ideal surfaces and high-vacuum conditions, which are typical for surface science studies. In addition, palladium can easily form carbide phase in the presence of hydrocarbons [29,31,32,33,34,35,36,37,38,39,40,41,42,43], which affects its catalytic properties. For this reason, the application of bulk-sensitive structural methods in addition to surface-sensitive ones is important to understand the structural evolution of the metal.

In a number of previous works, we have successfully applied in situ and operando X-ray absorption fine structure (XAFS) spectroscopy to follow the evolution of the catalyst under hydrogenation reaction conditions [32,33,36,38,44,45,46]. The extended XAFS (EXAFS) spectra provide direct information on the local atomic structure (interatomic distances and coordination numbers) around the element of interest (averaged over all of its possible locations). The X-ray absorption near-edge structure (XANES) is formed by the excitation of the core-level electron to the unoccupied states, being, therefore, sensitive to the electronic structure of material. This was efficiently used to discriminate palladium hydrides and carbides [32,36,38,47,48], which is usually a problem for hard X-ray-based techniques.

Here, we report the combined experimental and theoretical study of the industrial catalysts consisting of 2.6 nm supported palladium nanoparticles (NPs) probed by in situ EXAFS, XANES, X-ray diffraction (XRD) and diffuse reflectance infrared Fourier transform spectroscopy (DRIFTS), complemented by DFT calculations screening over a wide range of possible C_n_H_m_ adsorption intermediates. The use of complementary techniques allowed us to obtain a complete picture of the process, describing the evolution of both the structure of the palladium particles and ethylene molecules adsorbed on its surfaces. Beyond the standard fingerprint assignment of the spectroscopic features, XANES and infrared spectra were theoretically calculated based on DFT-relaxed atomic structure. This strategy allowed us to successfully follow the adsorption and dehydrogenation of ethylene on the surface of nanoparticles, with its further decomposition and formation of palladium carbide.

## 2. Materials and Methods

### 2.1. Materials

Commercial Pd/C and Pd/Al_2_O_3_ catalysts with average size of palladium particles was provided by Chimet S.p.A. The detailed characterization the catalysts and the support was performed elsewhere [49,50,51]. The metal loading was 5 wt.% for both samples and the average particle size was 2.6 nm with a small standard deviation of 0.4 nm. The choice of carbon support for synchrotron studies was explained by the possibility of XRD data collection for Pd phase, since in case of alumina support, the Pd reflections are overshadowed by those of alumina [52]. However, the carbon supported sample produced very poor DRIFTS signal; therefore, alumina support was chosen for infrared studies.

### 2.2. In Situ XAFS and XRD Measurements and Analysis

In situ Pd *K*-edge XAFS and XRD measurements were performed simultaneously for the Pd/C sample at BM31 beamline [53] of ESRF (Grenoble, France). The sample was loaded inside 2 mm quartz glass capillaries and connected to a remotely controlled gas line. The gas blower located under the sample was used to control the temperature. Prior to exposure to hydrocarbons, the samples were activated in 20 mL/min flow of 20% H_2_/He at 125 °C for 30 min and then purged by He flow. This procedure was to remove palladium oxides formed after continuous exposure to air and ensure the initial metallic state of palladium. Then, the sample was cooled down to 50 °C and exposed to pure ethylene flow (20 mL/min).

XAFS spectra were collected in transmission geometry using ionization chambers. The energy was selected by Si (111) double-crystal monochromator operated in continuous scanning mode and detuned to 80% of the maximum intensity to reduce the contribution of higher harmonics. For energy calibration, palladium foil was measured simultaneously with the sample using a 3rd ionization chamber. XRD patterns were collected in Debye–Scherrer geometry using a Dexcela CMOS 2D detector. The photon wavelength of 0.51105 Å was selected using a channel-cut Si (111) monochromator. The total time needed for one XAFS and XRD measurement was about 10 min.

XAFS data processing (energy calibration, normalization, background removal) and first-shell Fourier-analysis of EXAFS spectra were performed in Demeter software with standard parameters. The fit was performed independently for each spectrum using first-shell interatomic distance (*R*_Pd-Pd_), Debye–Waller factor (*σ*_Pd-Pd_), coordination number (*N*_Pd-Pd_), and zero energy shift (Δ*E*_0_) as fitting variables. Theoretical analysis of the XANES spectra was performed in PyFitIt code [54], which included principle component analysis (PCA) and fitting the experimental spectra by theoretical ones calculated within the finite difference method implemented in FDMNES code [55,56]. All calculations were performed with relativistic corrections and the radius of the computational sphere of 5.2 Å.

### 2.3. In Situ DRIFTS Measurements

In situ DRIFTS measurements for Pd/Al_2_O_3_ were performed on Vertex 70 spectrometer (Bruker, Billerica, MA, USA) equipped with a highly sensitive liquid mercury telluride detector. The choice of alumina support was determined by the better DRIFTS signal comparing to carbon support. The Praying Mantis low temperature reaction chamber (Harrick Scientific Products Inc., New York, NY, USA) was installed for DRIFTS measurements. Measurements were performed in range 5000–400 cm^−1^ with a 1 cm^−1^ resolution, 40 scans, and automatically transformed into absorption units using the Kubelka–Munk function. The self-written python code was then used to normalize the spectra by area and subtract the spectrum of activated state of the sample. The powdered sample (12.8 mg) was loaded into a cell enabling to control the temperature and the gas flow. An external gas system equipped with mass flow controllers (EL-FLOW, Bronkhorst High-Tech B.V., Ruurlo, Netherlands) was used to set the gas flows of Ar, H_2_, and C_2_H_4_ through the cell. The gas mixture flowed either through the cell with the sample, or through a by-pass. Switching was carried out using 3-way valves.

Similar to the procedure described in Section 2.2, the sample was activated for 30 min at 125 °C in 50 mL/min 5% H_2_/Ar and then cooled down to 50 °C (using liquid nitrogen as a refrigerant) and purged with inert gas (50 mL/min of Ar) to exclude the possible hydride phase. The lower hydrogen content compared to that used in Section 2.2, does not affect the finally obtained structure of metallic palladium. The higher values of gas flow were used due to the different geometry of the in situ reaction chamber compared to the capillary and bigger mass of the sample. The spectrum collected under such conditions was used as a background for the following spectra. The flow was then switched to 10% C_2_H_4_ in Ar and the spectra were collected continuously with the time-step of ca. 1 min. After the saturation in the spectra was observed, the sample was purged first by pure Ar and then exposed to 5% H_2_/Ar and purged again with Ar.

### 2.4. DFT Calculations

Atomic structures of the adsorbed C_n_H_m_ molecules on palladium (111) and (100) surfaces and their vibrational spectra were obtained using VASP 5.3 code [57,58] with PBE exchange-correlation potential [59]. Five layer Pd surfaces in (111) and (100) geometries were constructed with initial Pd–Pd distances *a* = 2.75 Å (that corresponded to the equilibrium unit cell parameter of the bulk structure) and then optimized within conjugated gradient algorithm. The surface approximation in periodic condition of VASP code was obtained by adding the vacuum above the top layer resulting the total height of the unit cell of 20 Å. The cut-off energy for the plane-wave basis set of 500 eV was used. The Monkhorst–Pack method was used to generate *k*-points 5 × 5 × 1 and 9 × 9 × 1 grids for (111) and (100) surfaces, respectively. The convergence criteria were set to 10^−6^ eV for self-consistent field calculations and 10^−5^ eV for geometry relaxation. The infrared spectra of the optimized structures with adsorbed hydrocarbon molecules were simulated keeping all palladium atoms fixed, which was checked not to affect the spectra for several selected cases. Calculation for an isolated ethylene molecule was performed in square 10 × 10 × 10 Å box with a single *k*-point. The above model does not consider the lower-coordinated palladium sites, which should be present on the surface of 2.6 nm NPs. However, this model was able to reproduce the experimentally observed features, which prevented us from its further complication.

## 3. Results

### 3.1. Evolution of the Bulk Structure of the NPs

**Figure 1:**
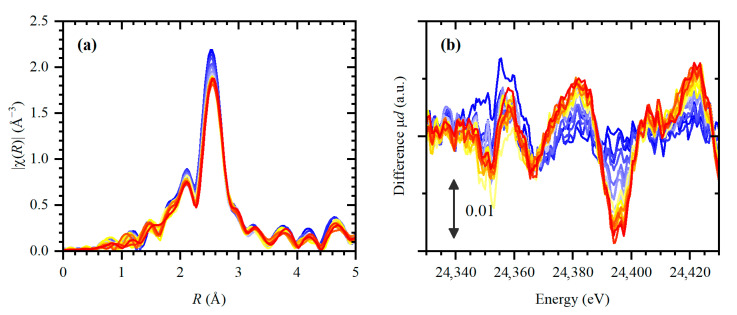
(**a**) Experimental FT-EXAFS spectra (phase uncorrected) and (**b**) difference XANES spectra (obtained by subtracting a spectrum of metallic Pd NPs) collected during exposure to ethylene at 50 °C (from blue to red) with the time step of ca. 10 min.

Evolution of EXAFS and XANES spectra collected during exposure of the sample to ethylene at 50 °C is shown in Figure 1 (parts a and b, respectively). The process was characterized by a slow and gradual shift of the first-shell peak in the Fourier transformed EXAFS (FT-EXAFS) data due to the expansion of palladium lattice. After almost 4 h of exposure to ethylene, Pd–Pd distances were increased by ca. 0.7% (in the fresh sample *R*_Pd-Pd_ = 2.736 ± 0.003 is close to previously reported values for metallic Pd NPs [43,50,52,60]). In addition, the intensity of the first shell peak decreases due to the increasing Debye–Waller factor (see Figure S1 and Table S1 in the Supplementary Information). Both observations are indicative of palladium carbide formation [34,36,38,40]. It should be noted that the coordination numbers are stable along the whole series of collected spectra indicating the stability of Pd particles (see Figure S1 and Table S1 in the Supplementary Information). The average value of *N*_Pd-Pd_ = 10 is consistent with 2.6 nm particle size.

**Figure 2:**
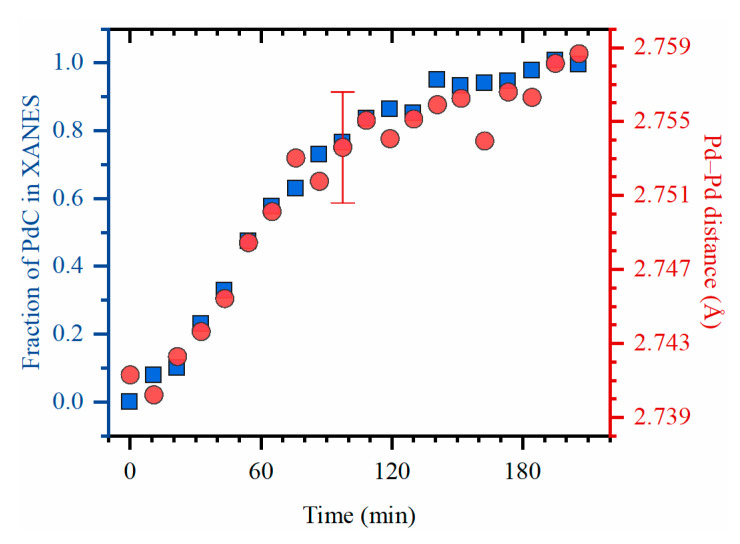
The results of LCF of XANES (blue squares, left ordinate axis) and first shell Pd–Pd interatomic distance obtained from EXAFS (red circles, right ordinate axis) for the experimental XAFS data collected during exposure to ethylene at 50 °C. The error bar corresponds to the maximal uncertainty in the fitted values of *R*_Pd-Pd_.

The changes in XANES are characteristic for palladium carbides [32] (see Section 3.3 for the interpretation), which supports the idea that the expansion of Pd lattice was induced by incorporation of carbon atoms into the octahedral interstitial sites. Results of linear combination fitting (LCF) of XANES data performed using the spectra of the metallic state and the sample after continuous exposure to ethylene and Pd–Pd interatomic distance obtained from EXAFS (Figure 2) demonstrate that the evolution of palladium structure proceeds in three steps: (i) “induction” period during the first ca. 20 min, (ii) fast evolution during the following 1 h, and (iii) the subsequent slower evolution.

The experimental spectral changes in XANES spectra reported in Figure 1b have two different origins. The changes in the higher energy region, starting from the peak at 24,380 eV are mainly influenced by the peak shift according to the Natoli rule due to the lattice expansion. In contrast, the region close to the edge position (see features A and B in Figure S2) is reflective of the new antibonding state forming due to the mixing of Pd–C orbitals. Since the observed lattice expansion is quite small (0.7%) compared to previously reported palladium carbides at higher temperatures [36,38], the expected carbon concentration in the bulk PdC_x_ is *x* ≈ 0.03 [61]. Therefore, the major contribution to the reshaping of near-edge structure is explained by surface adsorbed hydrocarbons. Figure S2 demonstrates that the experimental features can be reproduced by the model of di-σ-adsorbed-ethylene. Due to the fact that XANES signal is averaged over all atoms in the NPs, its sensitivity to the surface adsorbed species is significantly lower comparing to DRIFTS. Therefore, the discrimination of different surface species would be too ambiguous. However, the fact of Pd–C bonding is unambiguously proved. In addition, the analysis of Pd oxidation state performed by fitting the XANES spectra by those of bulk Pd metal and PdO references (see Table S1) reveals a small increasing trend from a Pd^2+^ fraction of 0.14 to 0.20, which is close to the experimental uncertainty of 0.04. The observed trend can also be explained by the fact that characteristic features of carbidic palladium in the region of the first XANES peak [32,34,39,60] are visually similar to those obtained by the addition of a PdO spectrum. The corresponding Pd^2+^ fraction for the fresh sample after activation in hydrogen is 0.16.

The averaged cell parameter obtained from XRD data was consistent with the *R*_Pd-Pd_ values from EXAFS. However, the evolution of XRD patterns shown in Figure S3 evidences that there is a stepwise change of the cell parameter due to the phase transition to α-carbide. This fact can be best appreciated on higher *hkl* reflections (e.g., 202 and 113, highlighted in Figure S2): the position of the metallic Pd peaks remains constant, while additional peaks appear at lower 2θ values.

### 3.2. Detection of Surface Species by In Situ DRIFTS

**Figure 3:**
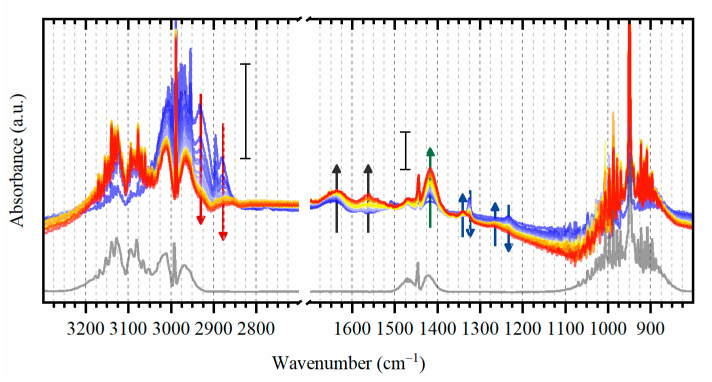
Evolution of experimental DRIFTS data (after normalization and subtraction of spectrum of the activated state) for Pd catalyst during exposure to ethylene at 50 °C (from blue to red) with the acquisition time of ca. 1 min. Grey line corresponds to the reference spectrum of gas-phase ethylene [71]. The scale bars demonstrate that the left part is enlarged with respect to the right one.

Upon exposure to ethylene, a number of bands are observed in DRIFTS spectra (Figure 3) with different positions with respect to gas-phase ethylene, which evidences its adsorption on palladium. In particular, in the high-frequency region corresponding to C-H stretching, the bands around 2900 cm^−1^ (shifted to the lower frequencies with respect to the gas phase ethylene) appear in the beginning of exposure and slowly decrease during the first 8 min (red arrows in Figure 3). This can be explained by the fact that ethylene initially adsorbed in di-σ configuration is then dehydrogenate to other intermediates (vide infra). The other bands in this region are also present but are significantly broadened which complicates their assignment. This may be due to the higher disorder in the stricture of 2.6 nm NPs in comparison with well-defined surfaces, and the presence of multiple species with overlapping C-H stretching modes. Therefore, the assignment below was made considering the 1000–1700 cm^−1^ region. Moreover, a weak band is observed at 2165 (see Figure S4 in the Supplementary Information), attributed to C≡C triple bond stretching.

The decreasing trend is also observed for two bands at 1325 and 1235 cm^−1^ with a parallel growth of the bands at 1340 and 1273 cm^−1^ (blue arrows in Figure 3). These features are not related to di-σ-adsorbed ethylene but can be explained by different frequencies (and intensities) which are expected for similar molecules on the surfaces with different interatomic distances (see Figure S7). Therefore, such behavior is related to the transition of palladium into its carbide phase with increased cell parameters as observed by EXAFS and XRD in Section 3.1.

**Figure 4:**
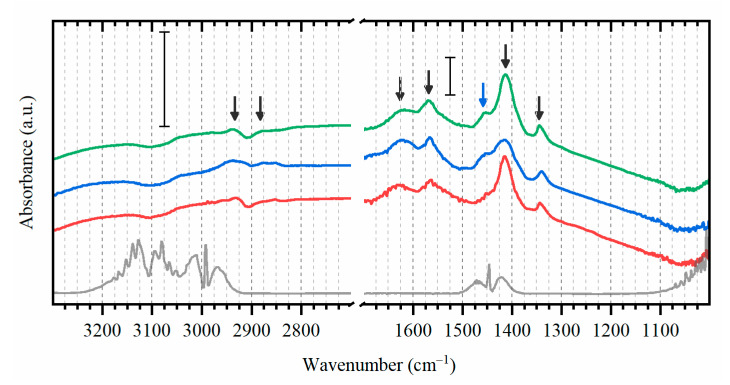
Background subtracted experimental DRIFTS data (after normalization and subtraction of spectrum of the activated state) for Pd catalyst after exposure to C_2_H_4_. The subsequent spectra were collected in argon (red), in hydrogen (blue) and again in argon (green). Black line corresponds to the reference spectrum of gas-phase ethylene [71]. For clarity, the spectra are shifted in vertical direction. The scale bars demonstrate that the left part is enlarged with respect to the right one.

The two growing peaks near 1600 cm^−1^ are characteristic for C = C double bond stretching. The one at a lower frequency appears with some delay with the respect to the higher one, and continues growing after ethylene was switched off (see Figure 4). The C = C bond can be explained by the formation of vinyl and vinylidene [16,62,63,64], the former also explains the peak at 1340 cm^−1^ discussed above related to C-H scissoring (see also Section 3.3). This peak was also reported for ethylidyne [12,14,16] but absence of C–C stretching near 1100 cm^−1^ allows us to exclude it.

The most intense peak observed at 1417 cm^−1^ (green arrow in Figure 3) is shifted to the lower frequencies with respect to C-H scissoring peak of gas phase ethylene. This band may be related to π- or di-σ-adsorbed ethylene [16,62,63,65,66], ethylidene [16,24,62,67], vinylidene [16] or methyl group (CH_3_) [19].

Most of the formed species remain stable not only upon purging with argon, but also after flowing hydrogen which is supposed to hydrogenate the surface adsorbed hydrocarbons (Figure 4). The addition of hydrogen leads to a formation of a distinct peak at around 1450 cm^−1^, which should be related to the partial hydrogenation of one of the adsorbed species. To highlight the effect of ethylene adsorption, the spectrum of fresh activated catalyst was subtracted from all the reported data. This procedure allowed excluding the vibrational frequencies related to the support itself and possible hydroxyl groups on alumina [68,69,70].

### 3.3. DFT Relaxation of C_n_H_m_ on Pd Surfaces and Their Vibrational Spectra

**Figure 5:**
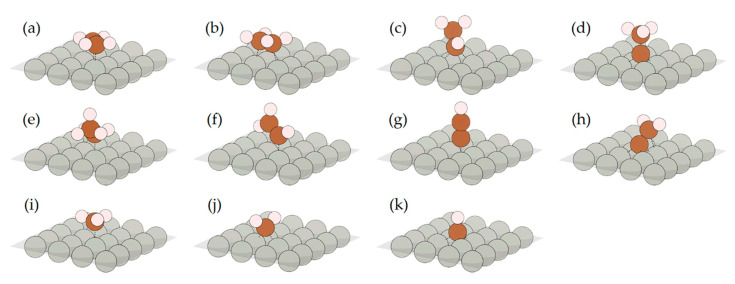
Visualization of the DFT-relaxed structures of different C_n_H_m_ species on Pd (111) surface: π- (**a**) and di-σ-adsorbed (**b**) ethylene, ethylidene (**c**), ethylidyne (**d**), ethyl (**e**), μ-vinyl (**f**), ethynyl (**g**). vinylidene (**h**), methyl (**i**), methylene (**j**) and methine (**k**). For better visualization, only the top surface layer of palladium is shown.

To correlate the experimentally observed features in XANES and DRIFTS data with the structure of the adsorbed species, we have considered a wide range of possible C_n_H_m_ molecules on the surface of palladium. For XANES calculation, the bulk PdC structure, with carbon atoms occupying octahedral interstitial sites of *fcc* lattice, was also considered. The DFT-relaxed atomic structures on Pd (111) are shown in Figure 5. For Pd (100), similar results were obtained, except for structures (d) and (k), due to the fact that there is no three-fold hollow site, and vinylidene (vide infra). The first two structures, (a) and (b), correspond to π- and di-σ-adsorbed ethylene, respectively. The two following structures are ethylidene (c) and ethylidyne (d), which were observed experimentally for different noble metal surfaces after interaction with ethylene [13,21]. The next three structures are on top adsorbed ethyl (e), μ-vinyl (f), and ethynyl (g). Vinylidene (h) was initially placed in bridge configuration orthogonal to the surface (μ-vinylidene). This configuration was preserved after relaxation for the surface of (100), but for the surface of (111), the μ_3_-η^2^-vinylidene was formed after relaxation which correlates with previous reports [21]. Finally, the C_1_-species (methyl (i), methylene (j) and methine (k) groups) were also considered to account for the possible decomposition of ethylene. In addition, π- and di-σ-adsorbed acetylene (C_2_H_2_) molecules were also relaxed, but are not shown in the Figure 5 since their formation after ethylene adsorption is the least expected.

The theoretical vibrational spectrum of an isolated ethylene molecule was in good agreement in both positions and intensities with the experimental data for gas-phase ethylene (see Section S5 in the Supplementary Information), which is an important prerequisite for the further comparison of theoretical and experimental spectra of unknown species. Below, the comparison of theoretical and experimental spectra is made based on both the absolute positions and the relative shifts with respect to gas-phase ethylene. The figures are reported in Section S2 of the Supplementary Information.

**Figure 6:**
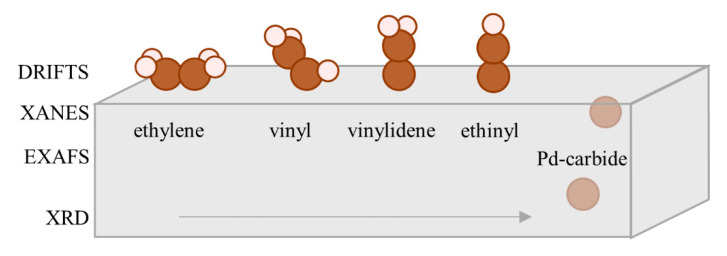
Ethylene dehydrogenation pathway based on XRD, EXAFS, XANES and DRIFTS data.

The shift of the C-H stretching bands of di-σ-adsorbed-ethylene (b) towards lower frequencies (Figure S6) supports the assignment of the decreasing bands near 2900 cm^−1^ made in Section 3.2. The C=C=C C stretching frequency of μ-vinyl is 60 cm^−1^ higher than in μ-vinylidene (Figures S7 and S8), which explains the two bands at 1627 and 1524 cm^−1^ in the experimental data (although the absolute values of theoretical frequencies are lower). The calculated frequency of C≡C bond of ethinyl (g) was also underestimated by almost 200 cm^−1^, which may be attributed to the higher interatomic distances (2.77–2.82 Å) compared to experimental EXAFS results (2.76 Å). In particular, in the presence of hydrogen, when the Pd-hydride is expected with increased Pd–Pd distances, the experimental C≡C band also shifts by more than 100 cm^−1^ to the lower frequencies (Figure S4b). The above structures demonstrate progressive dehydrogenation from C_2_H_4_ to C_2_H_1_ (Figure 6). As already mentioned, it is difficult to confirm or discard the presence of C_1_-species. The increase in the shoulder around 1450 cm^−1^ might be explained by hydrogenation of some part of the adsorbed species to ethyl (Figure S9).

## 4. Discussion

The synergetic coupling of EXAFS, XANES, XRD and DRIFTS data allows following the evolution of the bulk structure of palladium NPs and the speciation of C_n_H_m_ molecules at their surfaces in situ. Immediately after exposure to ethylene, the adsorption of gas phase ethylene on the Pd surface occurs, which does not induce any changes in the bulk structure of the NPs. The adsorbed ethylene is then partially converted to vinyl as of the stable intermediates. This process is accompanied with lattice expansion monitored by EXAFS, which means that the decomposition of ethylene to atomic carbon takes place. In this case, XANES acts as a bridge between EXAFS and DRIFTS, being sensitive to both lattice expansion and Pd–C bonding. With no evidence of palladium hydride formation, XANES supports the hypothesis that the observed lattice expansion is indeed due to the incorporation of carbon atoms to the bulk of NPs. As noted in Section 3.3, the region close to 24,350 eV mainly reflects formation of Pd–C bond. This explains the fact that the changes in this region occur almost immediately—since the main part of such bonds corresponds to the surface coverage of palladium by hydrocarbon molecules. In contrast, the changes in the higher energy region of XANES spectra are more gradual due to slow lattice expansion. Based on the correlation of DRIFTS peaks and the order of their appearance and the lattice expansion of the Pd lattice the dehydrogenation path ethylene → vinyl → vinylidene → ethinyl → palladium carbide can be suggested (Figure 6). In addition, it should be noted that the obtained hydrocarbons were stable under hydrogen flow. Considering the activity of palladium in hydrogenation reaction even at low temperatures [38], this result indicate that the revealed intermediates are the ones that are responsible for ethylene hydrogenation in catalytic reactions.

The observed decomposition of ethylene with the formation of carbide correlates with a number of previous reports [29,30,31,33,36,37,38,39,40]. The lattice expansion at 50 °C is, however, significantly reduced, in terms of both the kinetics and saturated values compared to the studies performed at higher temperatures [40]. The collection of high-quality synchrotron data was of utmost importance for highlighting these small changes. It should be noted that no evidence of ethylidyne formation was found, which was commonly observed during ethylene conversion over platinum [17,18,21,22]. A similar result was observed for the Pd (100) surface, at which ethylene dehydrogenates to vinyl without the formation of ethylidyne [63]. However, the main reason for the difference is the experimental conditions (atmospheric pressure and 50 °C temperature), under which the dehydrogenation and subsequent decomposition of ethylene to Pd carbide is the dominant reaction pathway, which is in correlation with a recent study by Jones et al. [29]. It should be noted that although having similar size distribution, the NPs proved by surface sensitive DRIFTS and bulk sensitive XANES/EXAFS/XRD techniques had two different types of supports—carbon and alumina, respectively—due to the features of the experimental techniques employed. We believe that the evolution of the catalyst and the substrate is similar with respect to the used techniques; however, one should keep in mind the role of the metal-support interaction [69,72], which may affect, in particular, the electronic state of palladium in these two cases, and lead to slightly different ethylene adsorption and dehydrogenation.

## 5. Conclusions

In conclusion, we have revealed the dehydrogenation of ethylene via vinyl, vinylidene and ethinyl to palladium carbide as the dominant reaction on the surface of 2.6 nm palladium NPs under atmospheric pressure and moderate temperature (50 °C). The combination of in situ XAFS, XRD and DRIFTS data provided not only complementary information, but also facilitated the mutual interpretation of the data from different techniques. This synergetic coupling, supported by theoretical simulations, was demonstrated to be an efficient approach for the in situ investigation of surface and bulk structures of palladium-based catalysts under reaction conditions.

## Supplementary Materials

The following are available online at https://www.mdpi.com/2079-4991/10/9/1643/s1, Table S1: Pd–Pd distances, Debye-Waller factors and coordination numbers obtained from EXAFS and the fraction of Pd^2+^ obtained by fitting the XANES spectra, Figure S1: Evolution of Debye-Waller factor and coordination number from EXAFS, Figure S2: (a) Experimental difference XANES spectrum obtained by subtraction the spectrum of metallic NPs and simulated ones. Part (b) shows the same experimental spectrum, but compared with simulations for the atom inside the bulk metallic Pd and bulk Pd carbide with increased cell parameter, Figure S3: XRD patterns collected during exposure to ethylene at 50 °C with the time step of ca. 10 min. Figure S4: (a) Background subtracted experimental DRIFTS data for Pd catalyst after exposure to C_2_H_4_. The subsequent spectra were collected in argon, in hydrogen and again in argon. Part (b) shows the time evolution of DRIFTS during hydrogen treatment, Figure S5: Experimental and theoretical spectra of ethylene (gas phase), Figure S6: Theoretical spectra of an isolated ethylene molecule and di-σ-adsorbed-ethylene on Pd (111), Figure S7: Theoretical spectra of an isolated ethylene molecule and μ-vinyl on Pd (111), Figure S8: Theoretical spectra of an isolated ethylene molecule and μ-vinylidene on Pd (100), Figure S9: Theoretical spectra of an isolated ethylene molecule and ethyl on Pd (111).
