# Supplementary material for: Dehydrogenation of Ethylene on Supported Palladium Nanoparticles: A Double View from Metal and Hydrocarbon Sides
Source: Nanomaterials (Basel). 2020 Aug 21;10(9):1643. doi: 10.3390/nano10091643 (PMC7560039; doi:10.3390/nano10091643)
Supplement: Supplementary file 1 [file nanomaterials-10-01643-s001.pdf]

nanomaterials

Supplementary Information

for

Dehydrogenation of Ethylene on Supported Palladium Nanoparticles: A Double View from Metal and Hydrocarbon Sides

Oleg A. Usoltsev [1], Anna Yu. Pnevskaya [1], Elizaveta G. Kamyshova [1], Andrei A. Tereshchenko [1], Alina A. Skorynina [1], Wei Zhang [2], Tao Yao [2], Aram L. Bugaev [1,*] and Alexander V. Soldatov [1]

[1] The Smart Materials Research Institute, Southern Federal University, 178/24 Sladkova, 344090 Rostov-on-Don, Russia; oleg-usol@yandex.ru (O.A.U.); annpnevskaya@yandex.ru (A.Y.P.); kamyshova.liza@gmail.com (E.G.K.); tereshch1@gmail.com (A.T.); alinaskorynina@gmail.com (A.A.S.); soldatov@sfedu.ru (A.V.S.)

[2] National Synchrotron Radiation Laboratory, University of Science and Technology of China, Hefei 230029, China; zwei2319@mail.ustc.edu.cn (W.Z.); yaot@ustc.edu.cn (T.Y.)

* Correspondence: abugaev@sfedu.ru; Tel.: +7-863-305-1996

1. Results of EXAFS analysis

The structural parameters extracted from the first-shell Fourier analysis of EXAFS are reported in Table S1 and visualized in Figure S5. Evolution of $R_{Pd\text{-}Pd}$ was reported in Figure 2 of the main text.

Table S1. Pd–Pd distances, Debye-Waller factors and coordination numbers obtained from EXAFS analysis (the typical uncertainties are 0.003 Å, 0.0004 Å^2, and 0.6, respectively) and the fraction of Pd^{2+} obtained by fitting the XANES spectra in 24330–24410 eV region by the spectra of bulk Pd metal and PdO references (the typical error is 0.04).

Scan number	$R_{Pd\text{-}Pd}$	$\sigma_{Pd\text{-}Pd}$	$N_{Pd\text{-}Pd}$	Pd^{2+} fraction
1	2.739	0.0074	10.1	0.14
2	2.741	0.0073	9.9	0.13
3	2.740	0.0072	9.7	0.14
4	2.742	0.0074	9.9	0.13
5	2.744	0.0076	9.8	0.13
6	2.745	0.0077	9.8	0.17
7	2.748	0.0076	9.6	0.14
8	2.750	0.0078	9.8	0.16
9	2.753	0.0081	9.8	0.14
10	2.752	0.0082	10.0	0.15
11	2.754	0.0079	9.5	0.15
12	2.755	0.0083	10.0	0.16
13	2.754	0.0084	10.0	0.16
14	2.755	0.0078	9.6	0.17
15	2.756	0.0085	10.1	0.16
16	2.756	0.0083	9.9	0.17
17	2.754	0.0084	10.1	0.16
18	2.757	0.0086	10.2	0.16
19	2.756	0.0084	10.0	0.17
20	2.758	0.0082	9.9	0.20

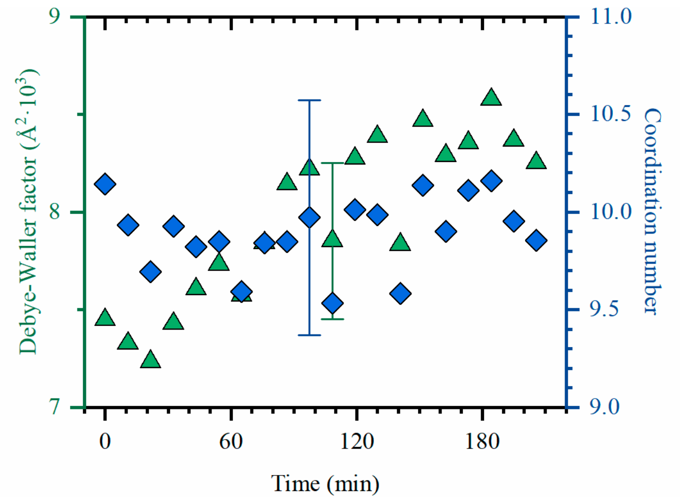

Figure S1. Evolution of Debye-Waller factor (**green triangles and left axis**) and coordination number (**blue diamond's and right axis**) extracted from EXAFS analysis.

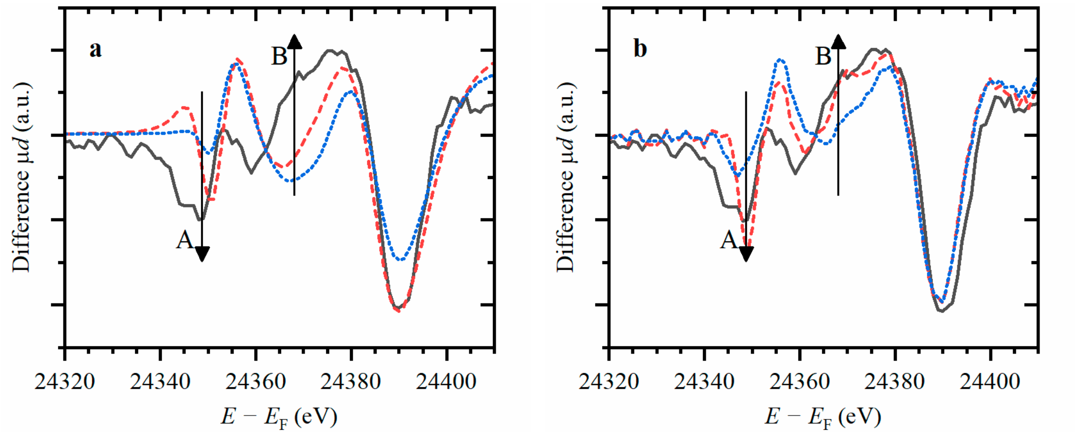

Figure S2. (**a**) Experimental difference XANES spectrum obtained by subtraction the spectrum of metallic NPs (solid black) and simulated ones using only the increased Pd-Pd distance (dotted blue) and with addition of di-σ-adsorbed-ethylene (dashed red). The simulations were done for Pd atom close to the adsorbed molecule. Part (**b**) shows the same experimental spectrum, but compared with simulations for the atom inside the bulk metallic Pd and bulk Pd carbide with increased cell parameter

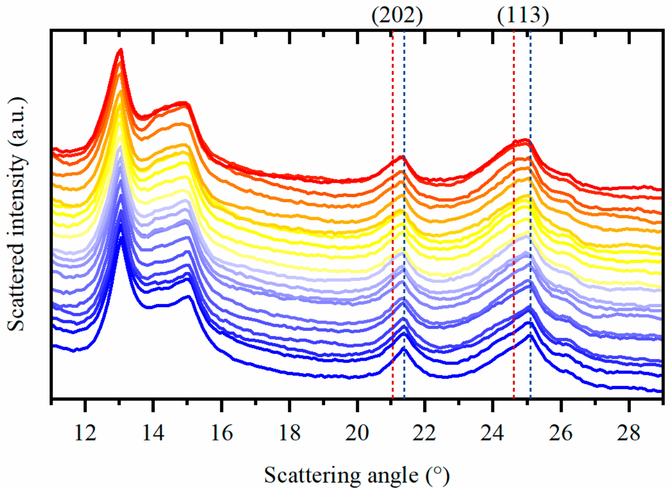

Figure S3. XRD patterns collected during exposure to ethylene at 50 °C (from blue to red) with the time step of ca. 10 min. Blue and red dashed lines highlight the peak positions of metallic palladium and palladium carbide, respectively.

2. Experimental and theoretical infrared spectra

Figure S4 shows the DRIFTS spectra in the region corresponding to C≡C triple bond stretching for the data presented in Figure 4 of the main text.

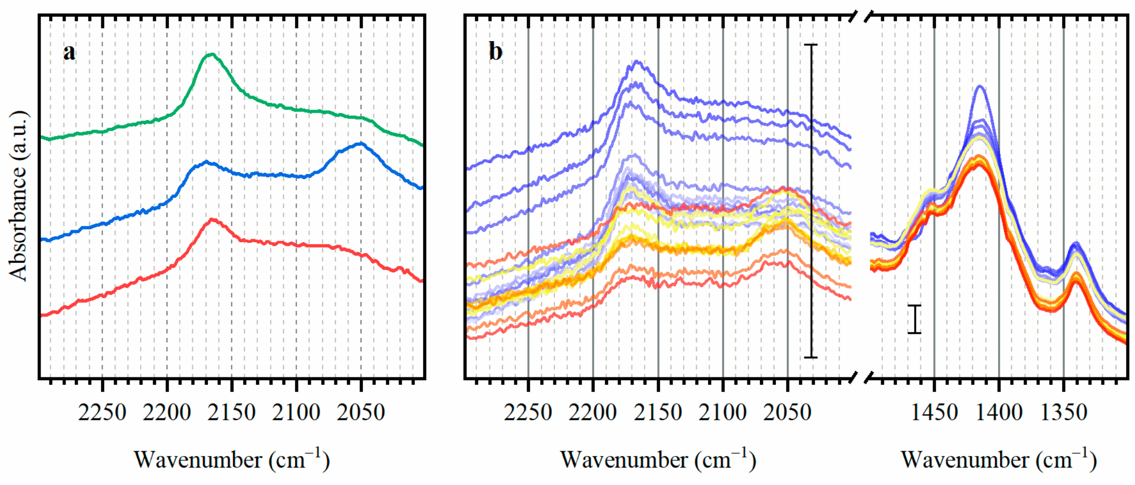

Figure S4. (**a**) Background subtracted experimental DRIFTS data for Pd catalyst after exposure to C_2H_4. The subsequent spectra were collected in argon (red), in hydrogen (blue) and again in argon (green). Part (**b**) shows the time evolution of DRIFTS during hydrogen treatment (no vertical shift applied).

The absolute values of theoretical vibrational frequencies often differ from the experimental ones. To estimate the effect of the chosen calculation parameters, we have computed the spectrum for an isolated ethylene molecule and compared it with the experimental spectrum of gas-phase ethylene [1]. As can be seen in Figure S5, the theoretical spectrum reproduce well both the positions and intensities of the experimentally observed peaks, which correspond to C-H stretching at 3168 and 3066 cm^{-1}, C–H scissoring at 1425 cm^{-1}, and C–H wagging at 939 cm^{-1} (the values correspond to the theoretical frequencies). Features around 1900 cm^{-1} represents second harmonics of 939 cm^{-1} peak that cannot be obtained in DFT simulation. However, for the stretching mode vibrations, the calculated frequencies are by 60–70 cm^{-1} higher than the experimental ones, which means that the relative shifts rather than absolute values should be considered for further interpretation in this region.

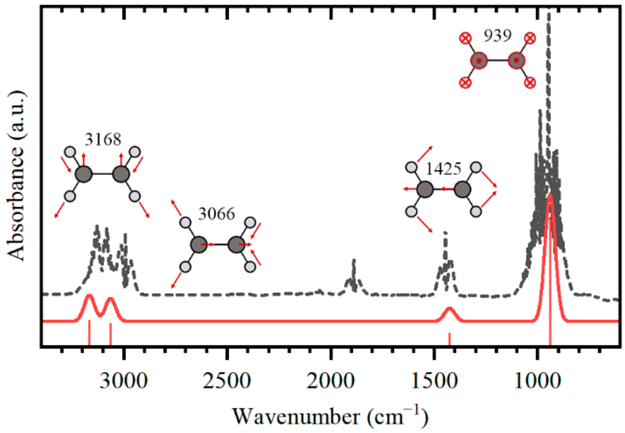

Figure S5. Experimental (**dashed black**) and theoretical (**solid red**) spectra of ethylene (gas phase).

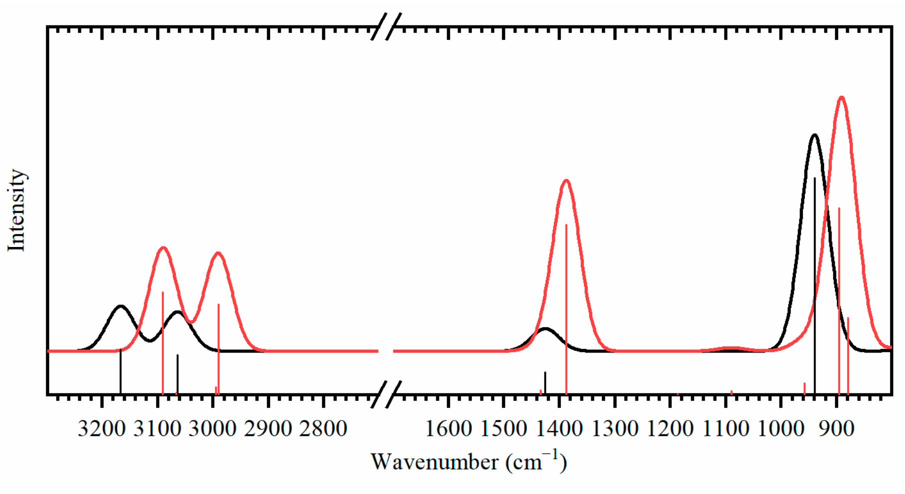

Figure S6. Theoretical spectra of an isolated ethylene (**black**) molecule and di-σ-adsorbed-ethylene (Figure 5b of the main text) on Pd (111) (**red**).

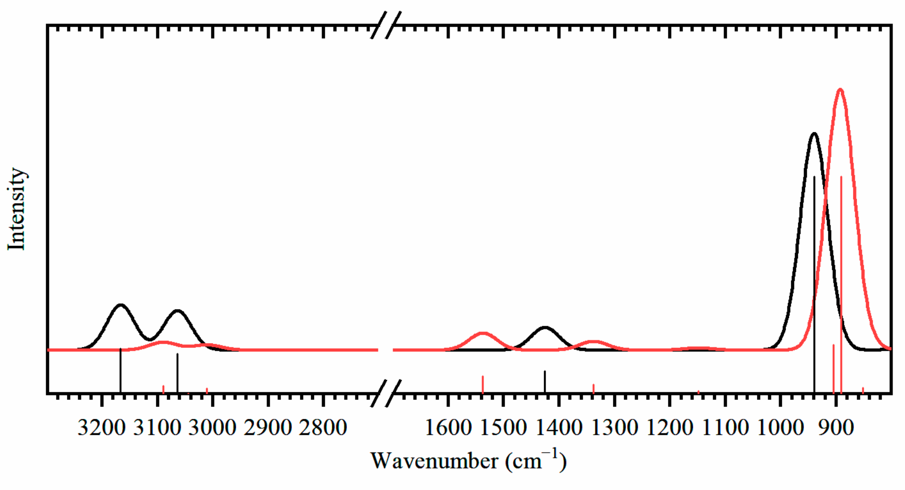

Figure S7. Theoretical spectra of an isolated ethylene (**black**) molecule and μ-vinyl (Figure 5f of the main text) on Pd (111) (**red**).

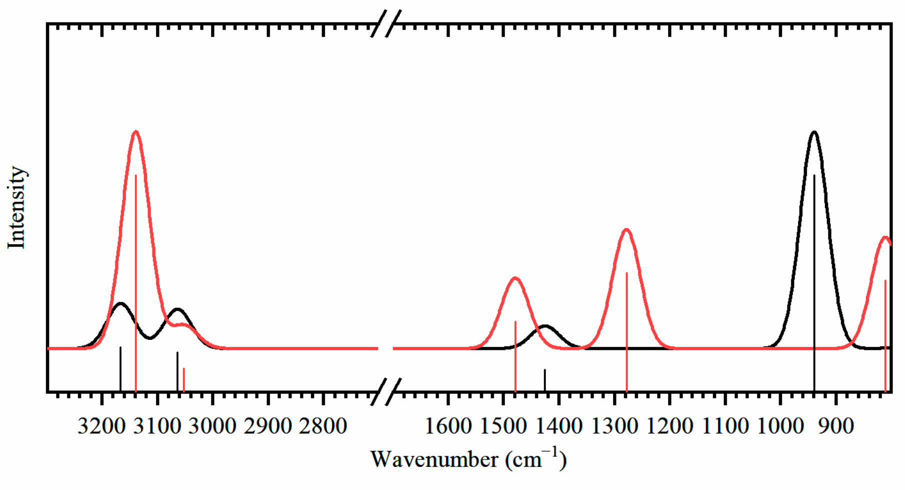

Figure S8. Theoretical spectra of an isolated ethylene (**black**) molecule and μ-vinylidene (Figure 5h of the main text) on Pd (100) (**red**).

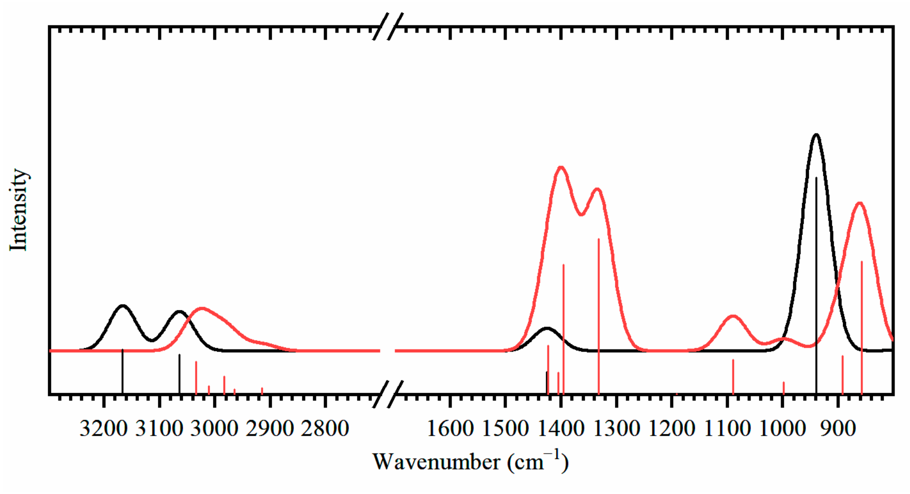

Figure S9. Theoretical spectra of an isolated ethylene (**black**) molecule and ethyl (Figure 5e of the main text) on Pd (111) (**red**).

References

1. Smith, A.L. The Coblentz Society Desk Book of Infrared Spectra. In *The Coblentz Society Desk Book of Infrared Spectra*; Carver, C.D., Ed.; Coblentz Society: Kirkwood, MO, USA, 1982; pp.1–24.

© 2020 by the authors. Licensee MDPI, Basel, Switzerland. This article is an open access article distributed under the terms and conditions of the Creative Commons Attribution (CC BY) license (http://creativecommons.org/licenses/by/4.0/).
